# The impact of pre- and postnatal exposures on allergy related diseases in childhood: a controlled multicentre intervention study in primary health care

**DOI:** 10.1186/1471-2458-13-123

**Published:** 2013-02-08

**Authors:** Christian Kvikne Dotterud, Ola Storrø, Melanie Rae Simpson, Roar Johnsen, Torbjørn Øien

**Affiliations:** 1 Department of Public Health and General Practice, Norwegian University of Science and Technology (NTNU), N-7489, Trondheim, Norway

**Keywords:** Asthma, Atopic, Dermatitis, Infant, Primary prevention, Public health

## Abstract

**Background:**

Environmental factors such as tobacco exposure, indoor climate and diet are known to be involved in the development of allergy related diseases. The aim was to determine the impact of altered exposure to these factors during pregnancy and infancy on the incidence of allergy related diseases at 2 years of age.

**Methods:**

Children from a non-selected population of mothers were recruited to a controlled, multicenter intervention study in primary health care. The interventions were an increased maternal and infant intake of n-3 PUFAs and oily fish, reduced parental smoking, and reduced indoor dampness during pregnancy and the children’s first 2 years of life. Questionnaires on baseline data and exposures, and health were collected at 2 years of age.

**Results:**

The prevalence of smoking amongst the mothers and fathers was approximately halved at 2 years of age in the intervention cohort compared to the control cohort. The intake of n-3 PUFA supplement and oily fish among the children in the intervention cohort was increased. There was no significant change for indoor dampness. The odds ratio for the incidence of asthma was 0.72 (95% CI, 0.55-0.93; NNT_b_ 53), and 0.75 for the use of asthma medication (95% CI, 0.58-0.96). The odds ratio for asthma among girls was 0.41 (95% CI 0.24-0.70; NNT_**b**_ 32), and for boys 0.93 (95% CI 0.68-1.26). There were no significant change for wheeze and atopic dermatitis.

**Conclusion:**

Reduced tobacco exposure and increased intake of oily fish during pregnancy and early childhood may be effective in reducing the incidence of asthma at 2 years of age. The differential impact in boys and girls indicates that the pathophysiology of asthma may depend on the sex of the children.

**Trial registration:**

Current Controlled Trials ISRCTN28090297.

## Background

The International Study of Asthma and Allergies in Childhood (ISAAC) has shown variations in the prevalence of allergy related diseases and that the worldwide time trend is increasing, particularly in developing countries [1,2]. Asher *et al.* suggested several factors to be involved, including lifestyle, dietary intake, microbial exposure, economic status, indoor and outdoor environment, climatic variation and awareness of disease [2]. The identification of which of these factors should be considered for early intervention and their effectiveness in counteracting the increasing incidence of allergy related diseases depends on the results of controlled intervention studies [3].

The Prevention of Allergy among Children in Trondheim (PACT) study was established to evaluate the possibility of reducing the prevalence of allergy related diseases in children within the constraints of public funding and time expenditure in primary health care [4]. Participants, regardless of parental history of atopy were included, as a large proportion of allergy related diseases develop in “low risk” groups [5,6], and “…a large number of people at small risk may give rise to more cases of disease than a small number who are at high risk” [7]. Smoking and environmental tobacco smoke (ETS) [8], oily fish, dietary n-3 polyunsaturated fatty acids (n-3 PUFAs) [9-11], and indoor dampness [12,13], were associated with the development of allergy related diseases and were of particular scientific interested when this study commenced. We have previously reported a significant reduction in parental smoking and increased intake of oily fish and n-3 PUFAs in the intervention cohort compared to the control cohort [4]. We have also shown a risk reduction from eating oily fish once a week or more at 1 year of age in terms of parental reported atopic dermatitis (AD) at 2 years of age [14]. The impact of altered exposure to these factors through a population based intervention on the incidence of allergy related diseases is unknown [15,16]. It is generally accepted that there are sex differences in the incidence of asthma in children, and studies which clarify the sex related factors in the development of asthma are needed [16].

The aim of the PACT study was to examine the feasibility and effectiveness of a non-targeted population-based intervention to decrease parental smoking and indoor dampness and to increase intake of oily fish and n-3 PUFA. The impact of the intervention was evaluated in terms of the prevalence of parentally-reported allergy related diseases at 2 years of age.

## Methods

### Design and participants

The PACT-study is an on-going controlled, multicentre interventional study, described in detail elsewhere [4]. Here we present the results from when the children were 2 years of age. The study was conducted in Trondheim, a city in the central part of Norway with 165 000 inhabitants and about 2100 births a year. The participants in both the control cohort and the intervention cohort followed the ordinary scheduled program for pre- and postnatal follow-up in primary health care.

The intervention cohort included women who presented to their GP or midwife for routine pregnancy check-ups after the implementation of the intervention in June 2002. Recruitment to the intervention cohort ended in May 2006. The control cohort recruitment commenced in September 2000 and consisted of pregnant women and children recruited at 6 weeks, 1 year and 2 years of age during routine check-ups with GPs and midwives across Trondheim. The recruitment of pregnant women to the control cohort ceased when recruitment into the intervention group commenced. When the first child in the intervention cohort was six weeks old, the recruitment of six-week-old children to the control cohort ceased, and analogously when they were 1 and 2 years of age. All women in the community who received an invitation to join the study, were willing and able to complete a questionnaire in Norwegian, and gave a written informed consent to participate were included. In this study, questionnaires on baseline data and all relevant exposures were collected during pregnancy and at 2 years of age. At the same time a questionnaire on the child’s health were collected, which was adapted to the age group from the ISAAC questionnaire [17], and tested for reliability in a separate study [18]. The health questionnaire focused on allergy related diseases and use of medication.

The intervention program was implemented by the City Government as a part of recommended maternity care and life-style counselling throughout the city, regardless of participation in the PACT-study or not. The smoking cessation intervention was adapted from the United States Department of Health and Human Services Public Health Service (USHPS) guideline "Treating Tobacco Use and Dependence” [19]. The dietary intervention was advice of intake of oily fish twice a week and 1.2 g n-3 PUFA a day (5 ml cod liver oil) during pregnancy. After birth intake of n-3 PUFA to the child was recommended from 4–6 weeks and intake of oily fish at least twice a week from 6 months of age. In the indoor dampness intervention, advice on how to detect housing dampness damage and how to reduce exposure to indoor dampness were given.

Parental smoking was assessed at 2 years of age through two questions asking the women if they, or their partner, were smoking at start of pregnancy, and if they were smoking now. Information on consumption of n-3 PUFA, lean fish (cod and coalfish) and oily fish (redfish, halibut, salmon, trout, herring and mackerel) as dinner and sandwich spread were collected using validated semi quantitative food frequency questions with six categories, and re-categorized later in the analyses [20,21]. Housing conditions and indoor dampness were assessed through the reported presence of eight different indicators of indoor dampness, including mouldy or musty smell, moist cardboard and newspapers after storage, dew on windows, moist spots on ceilings, walls or wallpapers, leakage detection on water pipes or faucet, leakage from roof or ground, or moisture in the floors. Dampness index was defined by the sum of reported dampness indicators with a sum ≥3 as the cutoff. In a separate study, information on education was collected from a sample of the control cohort and the intervention cohort by telephone interviews.

The primary outcome measure was based on a parental reported health questionnaire targeted to allergy related diseases. Allergic rhinoconjunctivitis (ARC) was not included as an outcome variable since incidence is low and an exact diagnosis of ARC is uncertain at 2 years of age. A child was considered to have asthma if the parents answered yes to “Has the child ever been diagnosed with asthma by a doctor?” Two questions in combination defined wheeze: “Has your child *ever* had whistling in the chest?” and “Has your child *ever* had episodes of wheezing or tightness in the chest?” Two questions in combination defined AD: “Has the child ever had eczema?” and “Has the child ever had an itchy rash which come and went over at least 6 months?” Parental atopy was defined as a history of asthma, ARC or AD in either the mother or father.

### Statistical analyses

The presentation of the results and interpretations follow the TREND recommendations for public health intervention research [22]. STATA (version 12.1 IC) was used for all statistical analysis.

Logistic regression was used to estimate crude odds ratios for each of the exposure variables and outcome variables in the intervention cohort compared with the control cohort. To estimate adjusted odds ratios (aOR) we used multilevel logistic regression models with a random intercept for each maternal health care centre. This model accounted for possible clustering of participants within different centres. All demographic variables (except education and parental smoking), and infectious diseases and use of antibiotics were considered as possible confounding factors, and were tested in separate multilevel logistic models for alteration on the effect estimator. In the final model, adjustments were made for birth weight, maternal atopy, and RS-virus infection. To adjust for possible between-centre confounders we adjusted for the mean number of participants from the intervention cohort within each maternal health care centre.

In a sub-analysis, the differential impact of sex on the behavioural and outcome measures were tested with an interaction term between sex and cohort, and further stratified by sex to obtain separate ORs for boys and girls. In a sub-analysis, the differential impact of parental smoking (mother or father at 2 years of age) on the outcome measures were tested with an interaction term between parental smoking and cohort, and further stratified by parental smoking to obtain separate ORs for asthma among children with smoking and non-smoking parents. In the trend analysis, we used binary logistic regression to estimate p-values for trend in both cohorts. In the analysis of the differences between the participants at 2 years of age and drop-outs in the prenatal questionnaire on exposures was used. For all other data, the questionnaires on exposures and health from 2 years were used. Two-sided significance tests were used, with p < 0.05 considered statistically significant. For interaction terms, p < 0.10 were considered statistically significant.

### Ethical approval

The study was approved by the Regional Committee for Medical Research Ethics for Central Norway (Ref. 120–2000). The Norwegian Data Inspectorate gave permission to process personal health data and one of the parents signed a written informed consent form (Ref 2003/953-3 KBE/-). The study is registered in Current Controlled Trials: ISRCTN28090297.

## Results

### Participants

**Figure 1:**
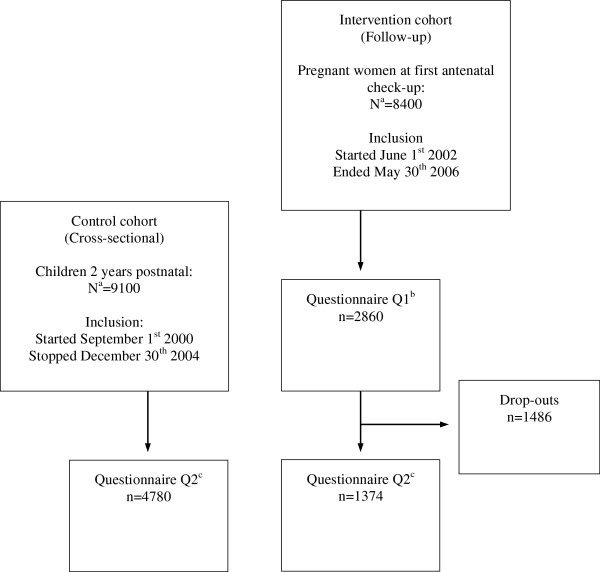
**Flow of participants in the intervention cohort and control cohort. **^a^ Total population of birth cohort in Trondheim during inclusion period. ^b^ Q1 = Completed questionnaire on exposures at first antenatal check-up during pregnancy. ^c^ Q2 = Completed questionnaires on exposures and health at 2 years of age.

Thirty-two of a total of 35 general practices, all seven community midwifes, and all 20 maternal health care centres recruited participants. The numbers of participants and time of inclusion are illustrated in Figure 1. The participants at 2 years of age in the intervention cohort and control cohort were comparable except from smoking status, single mothers and breastfeeding (Table 1).

**Table 1:** Demographic characteristics of participants at 2 years of age

**Characteristic**	**Intervention cohort N = 1374**	**Control cohort N = 4780**
	**Mean (SD)**	**Mean (SD)**
Age mother at delivery (years)	29.8 (4.21)	29.3 (4.68)
Education^a^ mother (years)	15.8 (2.43)	15.5 (2.58)
Education^a^ father (years)	15.1 (2.77)	15.0 (2.97)
Birthweight (grams)	3601 (545)	3582 (594)
	n (%)	n (%)
Boys	667 (49.3)	2389 (50.0)
One or more sibling	915 (67.4)	3265 (69.3)
Premature	162 (11.9)	671 (14.4)
Single mother	50 (3.8)	356 (7.7)
Breast fed ≥6 months	1053 (76.9)	3285 (69.3)
Smoking mother at start pregnancy	186 (13.8)	862 (18.6)
Smoking father at start pregnancy	205 (15.7)	917 (21.3)
Atopy^b^ mother	652 (47.8)	1991 (42.4)
Atopy^b^ father	578 (42.3)	1725 (36.8)
Atopy mother and father	304 (22.3)	865 (18.4)
Dog in dwelling	104 (7.6)	365 (7.7)
Cat in dwelling	150 (11.0)	397 (8.4)
Homeowner	1247 (91.4)	4118 (87.4)

^a^ Education data is based on a sample (Intervention cohort: n = 1062. Control cohort: n = 1242).

^b^ Defined as ever having had asthma, allergic rhinoconjunctivitis or AD.

In the intervention cohort, 2860 participants were included during pregnancy, and 1374 (48.0%) of these could be followed prospectively until 2 years of age. When comparing these participants (n = 1374) to the drop-outs (n = 1486), the age of the mothers were 29.3 (SD 4.18) and 28.5 (SD 4.60), the number of maternal smokers at start pregnancy were 257 (19.8%) and 345 (24.1%), the numbers of paternal smokers at start pregnancy were 223 (17.4%) and 335 (24.1%), and 1116 (83.2%) versus 1057 (70.7%) were homeowners, respectively. There were no differences between the participants and drop-outs regarding education, parity, parental atopy, and pets.

In the control cohort**,** 1796 pregnant women were included during pregnancy, and 833 (46.4%) of these could be followed prospectively until 2 years of age. When comparing these participants (n = 833) to the drop-outs (n = 963), the age of the mothers were 29.2 (SD 4.61) and 28.2 (SD 4.75), the number of maternal smokers at start pregnancy were 204 (25.1%) and 273 (29.5%), the numbers of paternal smokers at start pregnancy were 171 (21.9%) and 244 (27.7%), and 660 (79.8%) versus 652 (68.5%) were homeowners, respectively. Again, there were no differences between the participants and drop-outs regarding education, parity, parental atopy, and pets. Additionally, 3947 participants who were recruited to the control cohort after birth completed the 2 years questionnaire, giving a total of 4780 participants at 2 years.

### Behavioural changes

At 2 years of age, the prevalence of smoking amongst mothers (OR = 0.46; p < 0.01; Table 2) and fathers (OR = 0.57; p < 0.01) was almost halved in the intervention cohort compared to the control cohort. Intake of n-3 PUFA supplement was about 10 percentage points higher in the intervention cohort compared to the control cohort (OR = 1.46; p < 0.01). The proportion having oily fish at least once a week was about 15 percentage points higher in the intervention cohort (OR = 1.86; p < 0.01), and the intake of lean fish about 3 percentage points lower (OR = 0.87; p = 0.02). Indoor dampness index ≥3 was reported by approximately 4% of the participants in both cohorts (OR = 1.02; p = 0.90).

**Table 2:** Prevalence and odds ratios of exposures at 2 years of age

	**Intervention cohort N = 1374**	**Control cohort N = 4780**	**Odds ratio**^**a **^**(95%CI)**	
	**n (%)**	**n (%)**	**Crude**	**Adjusted**^**b**^
Smoking mother at 2 years of age	132 (9.8)	879 (19.0)	0.46	0.47
			(0.38-0.56)	(0.39-0.58)
Smoking father at 2 years of age	143 (11.2)	751 (17.9)	0.57	0.56
			(0.47-0.69)	(0.46-0.68)
Indoor dampness index	53 (3.9)	179 (3.8)	1.02	1.09
			(0.75-1.39)	(0.79-1.51)
1.2 g n-3 PUFA four times a week or more	582 (42.6)	1591 (33.6)	1.46	1.42
			(1.29-1.66)	(1.25-1.62)
Any kind of fish once a week or more	1011 (73.6)	3369 (70.6)	1.16	1.13
			(1.02-1.33)	(0.98-1.30)
Oily fish once a week or more	687 (50.0)	1667 (34.9)	1.86	1.83
			(1.65-2.11)	(1.61-2.09)
Lean fish once a week or more	836 (61.3)	3062 (64.6)	0.87	0.85
			(0.76-0.98)	(0.74-0.97)

^a^For intervention cohort versus control cohort.

^b^Adjusted for birthweight, maternal atopy, RS-virus infection, maternal health care centre, and the proportion of participants from each cohort in each public health centre.

### Incidence of allergic diseases

The incidence of parentally reported doctor diagnosed asthma in the intervention cohort compared with the control cohort was reduced (OR = 0.72; p = 0.01; Table 3). The absolute risk reduction (ARR) was 1.9% and numbers needed to treat to benefit (NNT_b_) was 53. The risk of using asthma medication during the last 12 months was correspondingly lower in the intervention cohort (OR = 0.75; p = 0.02). The differences between the intervention and control cohorts with respect to the risk of wheeze (OR = 0.92; p = 0.23) and AD (OR = 0.94; p = 0.47) were smaller and statistically non-significant.

**Table 3:** Incidence and odds ratios of allergy related diseases among 2 years old children

	**Intervention cohort N = 1374**			**Control cohort N = 4780**			**Odds ratio**^**a **^**(95%CI)**			
	**Boys n (%)**	**Girls n (%)**	**Total n (%)**	**Boys n (%)**	**Girls n (%)**	**Total n (%)**	**Boys crude**	**Girls crude**	**Total crude**	**Total adjusted**^**b**^
Asthma	55	16	71	208	129	337	0.93	0.41	0.72	0.68
	(8.1)	(2.3)	(5.2)	(8.7)	(5.4)	(7.1)	(0.68-1.26)	(0.24-0.70)	(0.55-0.93)	(0.52-0.90)
Used asthma medication last 12 months	60	19	79	222	138	360	0.95	0.46	0.75	0.69
	(8.9)	(2.7)	(5.8)	(9.3)	(5.8)	(7.5)	(0.70-1.28)	(0.28-0.74)	(0.58-0.96)	(0.53-0.91)
Wheeze	201	139	340	702	553	1255	1.02	0.82	0.92	0.91
	(30.7)	(20.4)	(25.4)	(30.3)	(23.9)	(27.1)	(0.84-1.23)	(0.66-1.00)	(0.80-1.05)	(0.79-1.06)
AD	109	107	216	427	360	788	0.87	1.02	0.94	0.93
	(16.5)	(15.8)	(16.1)	(18.5)	(15.5)	(17.0)	(0.69-1.10)	(0.81-1.30)	(0.80-1.11)	(0.78-1.10)
Ever used allergy medication	45	31	76	168	130	298	0.94	0.81	0.88	0.87
	(6.7)	(4.5)	(5.6)	(7.1)	(5.5)	(6.3)	(0.67-1.32)	(0.54-1.20)	(0.68-1.14)	(0.66-1.15)

^a^For intervention cohort versus control cohort.

^b^Adjusted for birthweight, maternal atopy, RS-virus infection, maternal health care centre, and the proportion of participants from each cohort in each public health centre.

Subgroup analysis stratified by sex disclosed a differential impact on asthma (p for interaction <0.01) and the use of asthma medication (p for interaction =0.01; Table 3). Among the boys, the impact on an asthma diagnosis was non-significant (OR = 0.93; p = 0.63), and correspondingly non-significant on asthma medication (OR = 0.95; p = 0.73). Among the girls, however, the impact on asthma (OR = 0.41; p < 0.01; NNT_**b**_ 32) and the use of asthma medication (OR = 0.46; p < 0.01) was statistically significant. Subgroup analysis stratified by sex showed a borderline non-significant differential impact of the intervention on wheeze (p for interaction 0.12). Among the boys, the impact on wheeze was non-significant (OR = 1.02; p = 0.85). While it was borderline non-significant for girls (OR = 0.82; p = 0.06), there was no differential impact of the intervention for AD in the subgroup analysis (p for interaction 0.34).

Subgroup analysis stratified on parental smoking at 2 years of age disclosed no differential impact of the intervention on asthma (p for interaction 0.71). The ORs for asthma in the intervention cohort compared with the control cohort among children with smoking parents (OR = 0.80; p = 0.45) and non-smoking parents (OR = 0.71; p = 0.02) were comparable.

### Other potential confounders

Comparisons of reported infectious disease and use of antibiotics among the children at 2 years of age are presented in Table 4. There was an increased risk of intestinal infection (p < 0.01), in the intervention cohort compared with the control cohort. There were no statistically significant differences between the intervention and control cohorts with respect to the risk of RS-virus infection, false croup, bronchitis, pneumonia, and the use of antibiotics. There were no statistically significant sex differences on infectious disease and use of antibiotics in either cohort (data not shown).

**Table 4:** Distribution of potential confounders of reported infectious disease and use of antibiotics among 2 years old children

	**Intervention cohort N = 1374**	**Control cohort N = 4780**	**Odds ratio**^**a**^
	**n (%)**	**n (%)**	**(95%CI)**
Ever RS virus infection	75 (5.5)	209 (4.4)	1.26 (0.96-1.65)
Ever intestinal infection	373 (27.2)	864 (18.1)	1.68 (1.46-1.94)
Ever false croup	188 (13.7)	675 (14.2)	0.96 (0.81-1.14)
Ever bronchitis	185 (13.5)	692 (14.6)	0.92 (0.77-1.09)
Ever pneumonia	87 (6.4)	345 (7.3)	0.87 (0.68-1.11)
Ever used antibiotics^b^	601 (44.0)	2196 (46.1)	0.92 (0.81-1.03)

^a^For intervention cohort versus control cohort.

^b^Ever used antibiotics for cold, inflammation of the ear, bronchitis, RS-virus infection, false croup, pneumonia, urinary infection and gut infection.

### Annual trend analysis

No statistical significant annual trends were found with respect to asthma in either the intervention cohort (OR = 0.92; p = 0.51) or the control cohort (OR = 0.97; p = 0.52). No statistical significant annual trends were found with respect to the use of asthma medicines in the intervention (OR = 1.07; p = 0.58) and the control cohort (OR = 1.04; p = 0.30).

## Discussion

We found a significantly lower incidence in parental-reported doctor diagnosed asthma and use of asthma medication at 2 years of age in the intervention cohort compared to the control cohort. The reduced incidence for both asthma and the use of asthma medication was observed among girls. There was no significant difference in the incidence of AD and wheeze between the intervention and control cohorts, and similarly no difference was observed when the groups were stratified by sex.

The behavioural changes are described in details elsewhere [4,23]. Briefly, the participants in the intervention cohort smoked significantly less, had an increased intake of oily fish and n-3 PUFAs, but the same levels of indoor dampness compared to participants in the control cohort.

As we did not find any difference between the cohorts regarding indoor dampness, the reduced incidence of asthma could not be ascribed to this interventional measure. The study was not designed to identify the magnitude of effect of individual interventions, and it remains therefore uncertain if the observed reduction in asthma is solely due to the reduction in tobacco smoke exposure or dietary alterations or a combination of both. Prenatal and postnatal smoke exposure is generally considered to be a strong risk factor for the development of respiratory symptoms and asthma, particularly in early life [24,25]. The first national comprehensive mass media campaign on tobacco and health for many years in Norway was accomplished during the study period in 2003, and a total ban on smoking in restaurants and cafés first took effect on June 1st 2004. According to WHO´ s European health for all database, the prevalence of regular daily smokers above 15 years of age in Norway has gradually declined during the study period, from 31.2% in year 2000 to 21% in 2008 [26]. In the sub-analysis of this study, the impact of the intervention among children with smoking and non-smoking parents was comparable. This may indicate that the reduced incidence of asthma in the intervention cohort was not a consequence of reduced parental smoking per se. Rather, reduced smoking in Norway in general and smoking bans in public places could have contributed to less ETS exposure of the children in the intervention cohort independent of the smoking status of their parents. In randomised controlled trials dietary supplementation with n3-PUFAs does not seem effective in reducing the incidence of asthma as showed in a meta-analysis [27]. However, fish intake during pregnancy and during the first year of life has been found to be associated with a reduced risk of childhood asthma [28-30]. Summarised, the body of evidence of a preventive effect of reduced ETS exposure on childhood asthma is stronger than the evidence of a preventive effect of fish. As such, the reduced incidence of asthma and use of asthma medication in the intervention cohort may be attributed first and foremost to the reduced ETS exposure of the children and secondly to the increased intake of fish. The trend analysis showed no annual trends in either cohorts regarding asthma. This indicates that the reduced risk of asthma in the intervention cohort was not a consequence of an annual time trend.

We found no differences between the cohorts regarding AD. We have previously reported a negative association between the intake of fish in childhood and AD at 2 years of age [14]. The increased fish consumption in the intervention cohort compared with the control cohort was likely too small to have an impact on AD on population level [4].

Our results demonstrated a strong differential impact on asthma and use of asthma medicines when stratified by sex. For wheeze, a similar differential impact was found, but was not statistically significant. An observational study has showed prenatal and postnatal exposure to tobacco smoke to be associated with asthma in 2 years old girls only [31], and cigarette smoking is associated with more respiratory symptoms among adolescent and adult women than among men [32,33]. A previous randomised intervention to reduce house dust mite, pet and food allergens, and passive smoking in a high-risk population of infants found an effect of the intervention on asthma-like symptoms in girls, but not on boys [34]. In line with this, our results indicate that the asthma phenotype among girls at 2 years of age is more susceptible to ETS and intake of oily fish than that of boys. The differential impact on boys and girls may indicate that the pathophysiology of asthma depends on the sex of the child.

The strengths of this study are the interventional, non-selected, population-based design with a large number of participants. The PACT study recruited participants from a primary health care population, regardless of parental history of atopy. The control cohort was established over a 2 year period immediately before the intervention was started, and from the same socio-economic areas. Participants in both cohorts followed the same scheduled program for pre- and postnatal follow-up in primary health care. Choosing a controlled design including whole birth cohorts made it possible to test the impact of the intervention program with a real life approach. A randomised control trial, with a co-existing cohort, would not be feasible in this type of broad population intervention, as both participants and health care workers move or communicate with each other introducing contamination of the control group. Secondly, comparing total birth cohorts also ensured high conformity between the cohorts regarding population size, race/ethnicity, maternal educational level, income, environment, urbanisation and social characteristics. The assessment of allergy related diseases were consistent through the observation period in both cohorts, and questions reporting factual information such as doctor diagnosed asthma were found reliable in a separate study [18].

Some 35% of the eligible pregnant women in Trondheim completed the questionnaires at 2 years of age. We had almost no active withdrawals in either cohort. The participation rate dropped during the study period [4], and could be a consequence of the long duration of the study resulting in a decreasing awareness, causing low recruitment activity among many GPs and midwifes, and not a consequence of self-selection among women. Decreased participation rate has been an increasing problem in epidemiological studies the recent years, but the participation rate experienced in this study might not introduce biased estimates if the participants are representative [35]. Results from the additional non-participants study (n = 391), showed no significant differences between non-participants and participants in the PACT study [23]. This is reassuring and indicates that the PACT population is comparable to the general maternal population in Trondheim.

The drop-outs in in the intervention cohort were different from the participants at 2 years of age regarding maternal age, maternal smoking at start pregnancy and homeowner status, but the clinical differences were small. Due to the design of the study, only a portion of the control cohort was followed prospectively from pregnancy, however similar differences were seen between the drop-outs and participants in the intervention and control cohorts.

The participation rate varied between each maternal health care centre and they had a somewhat different proportion of participants from each cohort. Multilevel logistic regression models compensated for possible clustering on maternal health care centre. The proportion of participants from each cohort in each public health centre was added as a separate variable to the model to compensate for the different participant rates from each cohort in each maternal health centre. We were able to adjust for a large number of confounders, including RS virus infection and other potential clinical confounders. Summarised in the final adjusted model, the aOR of allergy related diseases was comparable with the crude analysis (Table 3). This indicated that the effect estimates were not biased. We were unable to adjust for education due to lack of parental educational data on all participants. Parental homeowner status was tested in the adjusted model as a proxy for socioeconomic status, but did not alter the effect estimates, and was excluded in the final adjusted model.

## Conclusions

Reduced environmental tobacco smoke and increased intake of oily fish during pregnancy and early childhood may be effective in reducing the incidence of asthma at 2 years of age in primary health care. The differential impact in boys and girls indicates that the pathophysiology of asthma is dependent on the sex of the child.

## Competing interests

The authors declare that they have no competing interests.

## Authors’ contributions

RJ was responsible for the design, implementation of the trial, conduct, and contributed to analysis and interpretation and drafting the paper. TØ contributed to the design, implementation of the trial, validation and management of the main database, analysis and interpretation and drafting the paper. OS contributed to the design, implementation of the trial and drafting of the paper. CKD contributed to validation and management of the main database, was responsible for the analysis and interpretation, and was the principal author of the final report. MRS contributed to the interpretation of the analysis and drafting of the paper. All authors had full access to and can take responsibility for the data and analyses, and reviewed successive drafts of the paper. All authors read and approved the final manuscript.

## Pre-publication history

The pre-publication history for this paper can be accessed here:

http://www.biomedcentral.com/1471-2458/13/123/prepub
